# Determinants of Oral Hygiene Behaviours and Oral Health–Related Quality of Life Among Older Adults in Northern Thailand

**DOI:** 10.1016/j.identj.2025.109314

**Published:** 2025-12-10

**Authors:** Pechrada Wisespan, Penpatsson Promchat, Pornnapa Sukkho, Supang Wattanasoei, Supa Vittaporn, Patcharin Khamnuan, Surangrat Pongpan, Kasama Pooseesod, Sayambhu Saita

**Affiliations:** a Faculty of Public Health, Thammasat University, Lampang, Thailand; b Thammasat University Research Unit in Environment, Health and Epidemiology, Pathum Thani, Thailand; c Thammasat University Research Unit in One Health and Ecohealth, Pathum Thani, Thailand

**Keywords:** Denture, Oral health literacy, Oral hygiene, Oral health-related quality of life, Older adults

## Abstract

**Introduction:**

Oral health is an essential component of overall well-being, particularly among older adults. In Northern Thailand, where access to dental services varies by geography, older adult populations are at heightened risk for oral diseases, potentially compromising their quality of life. This study aimed to examine the determinants of oral hygiene behaviours and oral health–related quality of life (OHRQoL) among community-dwelling older adults in Northern Thailand.

**Methods:**

A cross-sectional analytic study was conducted between October and December 2024 in two districts of Lampang Province, representing urban and rural communities. A total of 240 participants aged ≥60 years were selected using multistage random sampling. Data were collected using structured interviews and clinical oral assessments. Key variables included sociodemographic, oral health literacy (OHL), self-care ability, denture use and number of remaining natural teeth. Oral hygiene behaviour was assessed with a 15-item WHO-guided questionnaire, while OHRQoL was measured using the Thai version of Oral Health Impact Profile instrument. Multivariable linear regression was used to identify significant determinants.

**Results:**

Factors significantly associated with better oral hygiene behaviour among older adults included those who were independent in daily life (*P* = .005), adequate OHL (*P* = .007) and denture use (*P* = .039). Denture use was also linked to better OHRQoL (*P* = .037).

**Conclusion:**

Findings highlight the importance of enhancing OHL, supporting dental care among dependent groups and ensuring access to appropriate prosthodontic care among the older adults. Tailored interventions should be developed to address these key determinants and improve oral health and quality of life in aging Thai communities.

## Introduction

Older adults experience disproportionately high burdens of oral disease worldwide, with dental caries prevalence ranging from 25% to 99% across populations and root caries affecting more than half of older adults in many regions.^1^ According to the 2022 World Health Organization (WHO) Global Oral Health Status Report, oral diseases impact more than 3.7 billion individuals aged 60 years and older, significantly contributing to disability‐adjusted life years in this age group.^2^, ^3^, ^4^ Similarly, national surveillance data from Thailand indicate substantial oral health challenges among older adults; findings from the Seventh Thai National Oral Health Survey revealed that approximately 60.60% of Thai adults aged 60 to 74 years report difficulties in chewing, a critical functional impairment that compromises nutrition, social interaction and overall quality of life.^5^

Preventive oral hygiene behaviours such as adequate tooth brushing, interdental cleaning and use of fluoridated toothpaste are essential for reducing the high burden of dental disease in older adults.^6^ However, adherence to oral hygiene behaviours remains inadequate among many older adults, influenced by multiple personal and contextual factors. In particular, oral health literacy (OHL) has emerged as a pivotal determinant of effective oral hygiene behaviours, older adults with limited OHL often struggle to adhere to recommended preventive practices due to difficulties understanding and acting on oral health guidance.^7^ In Thailand, nearly half of community‐dwelling elders possessed inadequate OHL, which was significantly associated with poorer oral hygiene practices.^8^

In addition to OHL, both self-care ability and clinical oral factors such as denture use and number of remaining teeth play significant roles in shaping oral hygiene practices, which subsequently influence oral health-related quality of life (OHRQoL) in older adults. Self‐care ability, encompassing both physical and cognitive capacities required to perform daily tasks, has been consistently associated with oral hygiene routines, particularly among dependent elders who face difficulties performing routine oral hygiene practices.^9^^,^^10^ Furthermore, clinical oral factors such as denture use and the number of remaining natural teeth play an essential role in maintaining oral function, particularly mastication, and may encourage better adherence to oral hygiene practices.^11^^,^^12^

Effectively addressing oral health issues among older adults requires comprehensive public health strategies that reflect the complexity of influencing factors at both individual and health service levels. Understanding the interplay of various determinants including sociodemographic, self-care ability, OHL and clinical oral factors can inform more targeted and culturally appropriate oral health promotion initiatives. Such efforts are essential for enhancing the oral health and overall quality of life of older adults.^7^

Despite growing recognition of the importance of oral hygiene behaviours and OHRQoL in older adults, existing research has largely examined these outcomes in isolation or narrowly defined contexts. Prior studies have typically focused on single determinants such as OHL or tooth number without considering broader factors like sociodemographic, self-care ability and denture status in a comprehensive model.^13^, ^14^, ^15^ Furthermore, evidence on how these determinants interact to influence both oral hygiene behaviours and perceived oral health outcomes remains limited, particularly among community-dwelling older adults in geographically diverse regions such as Northern Thailand. While national surveys have reported high burdens of oral disease among Thai older adults,^16^ few investigations have explored rural-urban disparities or adopted a multidimensional framework for analysis.^17^^,^^18^

To address these knowledge gaps, this study concurrently examined sociodemographic characteristics, OHL, self-care capacity and clinical oral factors in relation to both oral hygiene behaviours and OHRQoL among community-dwelling older adults in Northern Thailand, a region marked by well-documented inequalities in access to dental care. This investigation was guided by a conceptual model in which OHL, self-care ability and clinical factors (denture use, number of remaining teeth) directly influence oral hygiene behaviours, which in turn shape OHRQoL. This study hypothesised that older adults with higher OHL, greater self-care independence and better clinical oral factors would exhibit better oral hygiene behaviours and report higher OHRQoL. The findings may offer context-specific evidence to inform the design of integrated, community-based oral health interventions tailored to aging populations in underserved regions.

## Methodology

### Study design and study areas

This analytic cross-sectional study was carried out from October to December 2024 in two purposively selected districts of Lampang Province, Northern Thailand, chosen to reflect divergent community contexts. Mueang Lampang District, the provincial capital, exemplifies an urban setting characterised by well-developed infrastructure, including tertiary hospitals, private dental clinics and dense transport networks. In contrast, Mae Mo District represents a rural environment where the local economy is dominated by the Electricity Generating Authority of Thailand’s lignite mine and power plant. Dental care in Mae Mo is principally provided through primary health centres and mobile outreach units under the Ministry of Public Health, with service delivery challenged by geographic dispersion and lower population density (Figure).

**Figure:**
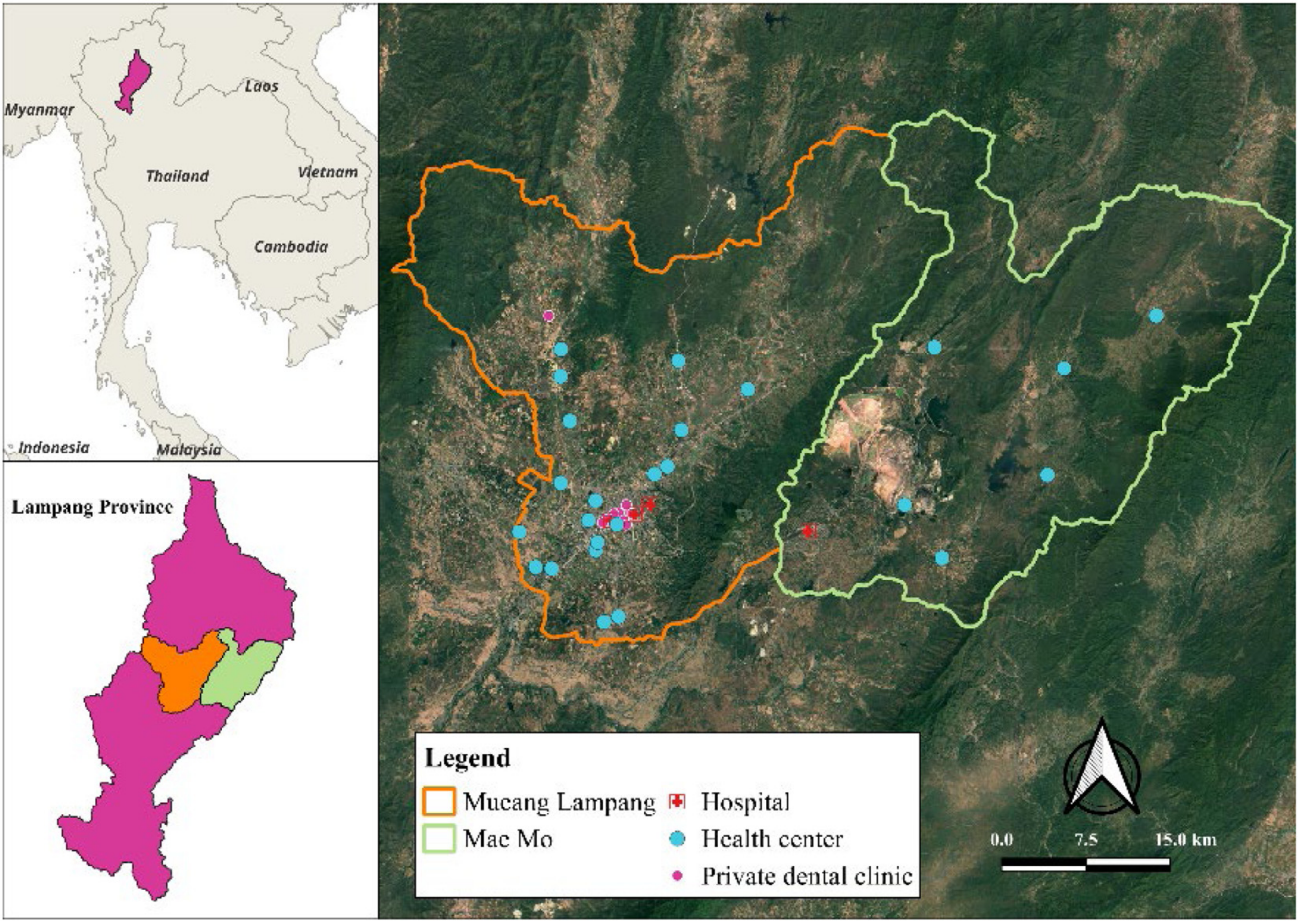
Distribution of oral-health service facilities in Mueang Lampang and Mae Mo Districts, Lampang Province, Thailand.

### Participants

A priori power analysis was performed using G*Power version 3.1.9.7 to estimate the required sample size for detecting significant associations with OHRQoL, the primary study outcome. Using a fixed-model F-test for linear multiple regression, we assumed a medium effect size (Cohen’s f² = 0.15),^19^ a significance level of α = 0.05, statistical power of 0.95, and 15 predictors: study area, sex, age, BMI, education, marital status, living arrangement, chronic disease, alcohol use, smoking, self-care ability, number of remaining natural teeth, denture use, oral health literacy and oral hygiene behaviours. The required minimum sample size was 199 participants. Based on prior research reporting a response rate of 74.3% for older adults.^20^ After adjusting for an anticipated nonresponse rate of 25%, the required sample increased to 249.

A multistage sampling method was employed to recruit participants for this study. Initially, two districts in Lampang Province, Mueang Lampang (urban) and Mae Mo (rural), were purposively selected. Subsequently, one subdistrict was randomly chosen from each district (Bo Haeo Subdistrict in Mueang Lampang and Mae Mo Subdistrict in Mae Mo). Within each selected subdistrict, simple random sampling with probability proportional to size was used to determine the number of participants, 149 individuals from Bo Haeo and 100 individuals from Mae Mo, yielding a total sample of 249 participants. Eligible participants were community‐dwelling residents aged 60 years or older who were able to communicate verbally and provided informed consent. Exclusion criteria comprised conditions that could impede questionnaire completion (eg, severe orofacial pain), critical physical illnesses, cognitive impairments (such as dementia or major psychiatric disorders), oral cancer and incomplete survey responses.

A total of 249 older adults were invited and 246 agreed to participate. After excluding 4 participants with incomplete questionnaires and 2 with inconsistent data, 240 complete cases were included in the final analysis.

### Variables and measures

#### Dependent variables

A 15-item oral hygiene behaviour questionnaire was developed based on recommendations from the World Health Organization Oral Health Survey Guidelines^21^ (Additional file 1). The items were designed to reflect key domains of daily oral health practices relevant to older adults. Items reflecting positive practices were rated on a three‐point Likert scale (1 = never; 2 = sometimes; 3 = always), while negatively worded items were reverse‐scored, yielding a total score range of 15 to 45 (higher scores denote more favourable behaviours). Individual item means were classified according to Best’s criteria^22^ including low (1.00-1.69), moderate (1.70-2.39) and good (2.40-3.00). The questionnaire underwent content validity assessment by three experts in epidemiology, dental public health and geriatric care. The Index of Item-Objective Congruence value ranged from 0.80 to 1.00, indicating acceptable to excellent agreement. Internal consistency reliability was confirmed with a Cronbach’s alpha coefficient of 0.82.

OHRQoL was measured using the validated Thai Oral Health Impact Profile instrument 14 items (OHIP-14),^23^ encompassing seven impact dimensions (functional limitation, pain, psychological discomfort, physical disability, psychological disability, social disability, handicap). Respondents rate the frequency of each impact on a five-point scale (0 = never to 4 = very often), yielding a total score range of 0 to 56, with higher scores reflecting greater negative effects on quality of life.

#### Independent variables

Socio‐demographic data were obtained via structured interviews and included study area, age, sex, body mass index (BMI), educational attainment, marital status, living arrangement, presence of chronic disease, current smoking status and alcohol consumption.

Self-care ability was assessed by self‐reported independence in activities of daily living (ADLs). Participants who were fully independent in all ADLs were classified as self‐care able, whereas those requiring assistance with one or more ADLs were classified as dependent.

Clinical oral factors were operationalised using two measures: denture use and the number of remaining natural teeth. Denture use was ascertained by participant self‐report and categorised dichotomously as non‐users or users of dentures. The count of remaining natural teeth was obtained through a guided self‐examination, with participants recording the total number of teeth present.

OHL was assessed using the Older Adults–Test of Functional Health Literacy in Dentistry (OA-TOFHLiD), which comprises 39 reading-comprehension items and nine numeracy items tailored to dental contexts. Total scores range from 0 to 48, with values of 41 or higher denoting adequate literacy. In its Thai validation among community-dwelling elders, the OA-TOFHLiD demonstrated good internal consistency with a Cronbach’s α = 0.87.^24^

### Ethical approval

The study protocol was conducted in accordance with the Declaration of Helsinki and was approved by the Institutional Review Board of Boromrajonani College of Nursing, Nakorn Lampang, Thailand (No. E2567-058 with a date of approval of 19 September 2024).

### Statistical analysis

All data were entered into and validated in Microsoft Excel before analysis in STATA version 15 (licensed to the Faculty of Public Health, Thammasat University, Thailand). Descriptive statistics including means and standard deviations for continuous variables, and frequencies and percentages for categorical variable were computed for the total sample. Univariable linear regression models were first fitted to screen for factors associated with oral hygiene behaviour scores and OHIP-14 scores. Variables with *P* < .20 in univariable analysis together with a priori covariates (study area, sex and age group) were then included in separate multivariable linear regression models to identify independent predictors of each outcome. Statistical significance was defined as *P* < .05.

## Results

### Characteristics of participants

The study comprised 240 participants, predominantly female (62.08%), aged between 60 and 69 years (70.83%), with a mean age of 67.23 ± 6.21 years. The majority of participants had a normal BMI (40.83%), whereas 24.17% were classified as having obesity class I, with a mean BMI of 23.90 ± 3.74. Regarding education, 77.92% attained an education level of primary or lower. A majority were married (67.08%) and resided with family members (91.67%). Chronic conditions were present in 59.58% of participants. Behavioural patterns revealed that 65.42% were people who abstain from alcohol, whereas 81.67% were nonsmokers. A significant majority exhibited proficient self-care capabilities (96.67%). Regarding dental health, individuals possessed an average of 24.39 ± 8.73 remaining natural teeth, with 50.42% maintaining 20 to 29 teeth. About 17.50% of interviewees reported using dentures (Table 1).

**Table 1:** General characteristics and clinical oral factors.

Characteristics	n	%
Sex		
Female	149	62.08
Male	91	37.92
Age (years)		
60-69	170	70.83
70-79	55	22.92
≥80	15	6.25
Mean ± SD	67.23 ± 6.21	
BMI group		
Normal (18.6-22.9)	98	40.83
Underweight (≤18.5)	12	5.00
Overweight (23.0-24.9)	53	22.08
Obesity Class 1 (25.0-29.9)	58	24.17
Obesity Class 2 (≥30)	19	7.92
Mean ± SD	23.90 ± 3.74	
Education attainment		
Primary education or lower	187	77.92
Secondary education or higher	53	22.08
Marital status		
Married	161	67.08
Single	17	7.08
Widowed/divorced	62	25.83
Living arrangement		
Living with family	220	91.67
Living alone	20	8.33
Chronic diseases		
No	97	40.42
Yes	143	59.58
Alcohol consumption		
Nondrinker	157	65.42
Drinker	83	34.58
Smoking		
Nonsmoker	196	81.67
Former or current smoker	44	18.33
Self-care ability		
Self-care able	232	96.67
Dependent	8	3.33
Number of remaining natural teeth		
≥30	76	31.67
20-29	121	50.42
10-19	14	5.83
0-9	29	12.08
Mean ± SD	24.39 ± 8.73	
Use of dentures		
No	198	82.90
Yes	42	17.50

### Oral health literacy

The assessment of OHL among older adult participants revealed an average total OHL of 34.76 ± 7.29 out of a total 48 points. The scores for reading comprehension and numeracy/text interpretation were 28.22 ± 6.28 and 6.54 ± 2.18, respectively. When categorised the level of OHL, 24.58% of participants demonstrated adequate OHL, while the majority (75.42%) had inadequate OHL (Table 2).

**Table 2:** Level of oral health literacy.

Oral health literacy elements	Mean ± SD
Reading comprehension	28.22 ± 6.28
Numeracy and text interpretation	6.54 ± 2.18
Total score	34.76 ± 7.29
Oral health literacy level, n (%)	
Adequate (OA-TOFHLiD ≥ 41)	59 (24.58)
Inadequate (OA-TOFHLiD < 41)	181 (75.42)

### Oral hygiene behaviours

The study assessed oral hygiene behaviours across 15-item oral hygiene behaviour questionnaire. The mean total score was 37.36 ± 3.16. Key behaviours performed well included refrain from smoking (2.89 ± 0.43), brushing teeth with fluoride toothpaste (2.86 ± 0.44) and avoiding sharing toothbrushes (2.90 ± 0.44). However, low compliance was noted in the use of dental floss (1.54 ± 0.74) and moderating behaviours such as brushing forcefully (2.28 ± 0.78), delaying treatment for visible dental issues (2.23 ± 0.77), or consumption of sweet or sticky snacks between meals (2.06 ± 0.67) (Table 3).

**Table 3:** Mean and standard deviation of item-level scores from the 15-item oral hygiene behaviour questionnaire.

No.	Item	Mean ± SD	Level
1	Brush your teeth for at least 2 min each time.	2.74 ± 0.51	Good
2	Use your own toothbrush and do not share it with others.	2.90 ± 0.44	Good
3	Replace your toothbrush when the bristles become frayed or splayed.	2.70 ± 0.60	Good
4	Clean your teeth using dental floss.	1.54 ± 0.74	Low
5	Brush your teeth gently with the correct technique.	2.28 ± 0.78	Moderate
6	Use toothpaste containing fluoride when brushing.	2.86 ± 0.44	Good
7	Use appropriate tools (not your teeth) to open packaging or bottles.	2.83 ± 0.48	Good
8	Visit a dentist when you experience a toothache.	2.25 ± 0.71	Moderate
9	Seek treatment promptly when cavities or tooth decay are noticed.	2.23 ± 0.77	Moderate
10	Visit a dental professional when abnormalities in your mouth are detected.	2.42 ± 0.72	Good
11	Limit consumption of sweet or sticky snacks between meals.	2.06 ± 0.67	Moderate
12	Refrain from smoking to promote oral health	2.89 ± 0.43	Good
13	Choose water instead of soda, sugary drinks, tea, or coffee.	2.58 ± 0.61	Good
14	Check your gums and teeth regularly for cleanliness and abnormalities.	2.53 ± 0.67	Good
15	Maintain regular oral hygiene practices every day.	2.55 ± 0.63	Good

Note: Values represent the mean ± SD of individual item scores within each behavioural subdomain, rated on a 3-point scale (1 = never, 2 = sometimes, 3 = always). Higher scores indicate more favourable oral hygiene behaviours.

### Oral health–related quality of life

The study revealed various dimensions of OHIP. The average overall impact score was 9.09 ± 7.96. The highest scores were observed in physical pain, highlighting the prevalence of issues like difficulty eating (1.13 ± 1.27) and oral pain (0.87 ± 0.99). The functional limitation also showed notable effects, with difficulties in taste perception (0.76 ± 1.06) and pronunciation reported (0.57 ± 0.96). Psychological and social impacts, though less pronounced, indicated concerns about self-image (0.69 ± 1.08) and reduced life satisfaction (0.60 ± 0.98) (Table 4).

**Table 4:** Oral health impact profile scores.

Dimensions	Mean ± SD
Functional limitation	
1. Difficulty pronouncing certain words	0.57 ± 0.96
2. Deterioration in taste perception	0.76 ± 1.06
Physical pain	
3. Pain in the mouth	0.87 ± 0.99
4. Difficulty eating	1.13 ± 1.27
Psychological discomfort	
5. Concern about self-image	0.69 ± 1.08
6. Feeling stressed	0.45 ± 0.85
Physical disability	
7. Dissatisfaction with eating	0.75 ± 1.02
8. Stopping meals midway	0.60 ± 0.94
Psychological disability	
9. Feeling uneasy	0.49 ± 0.82
10. Feeling embarrassed due to dental issues	0.57 ± 1.05
Social disability	
11. Irritation towards others	0.62 ± 0.89
12. Difficulty performing routine tasks	0.46 ± 0.84
Handicap	
13. Reduced life satisfaction	0.60 ± 0.98
14. Inability to perform tasks effectively	0.54 ± 0.89
Overall oral health impact profile	9.09 ± 7.96

### Factors associated with oral hygiene behaviour and OHIP scores

Multivariable linear regression identified three significant factors associated with oral hygiene behaviour scores including self-care ability, OHL and denture use. Participants who were independent in daily living had significantly higher oral hygiene behaviour scores than those requiring assistance (β = 3.36, 95% CI: 1.04-5.67, *P* = .005). Similarly, individuals with adequate OHL scored higher than those with inadequate literacy (β = 1.31, 95% CI: 0.36-2.26, *P* = .007). Denture wearers also exhibited slightly higher oral hygiene behaviour scores compared to nonwearers (β = 1.02, 95% CI: 0.05-1.98, *P* = .039). Additionally, denture use was the only factor significantly associated with better OHRQoL, reflected by lower OHIP scores (β = −2.61, 95% CI: −5.05 to −0.16, *P* = .037) (Table 5).

**Table 5:** Factors associated with oral hygiene behaviour and OHIP scores.

Factors	Oral hygiene behaviour			OHIP		
	β*	95% CI	*P*	β†	95% CI	*P*
Self-care ability						
Dependent	0 (ref.)					
Self-care able	3.36	1.04 to 5.67	.005			
OHL level						
Inadequate	0 (ref.)					
Adequate	1.31	0.36 to 2.26	.007			
Use of dentures						
No	0 (ref.)			0 (ref.)		
Yes	1.02	0.05 to 1.98	.039	−2.61	−5.05 to −0.16	.037

⁎ Adjusted for study area, sex, age group, educational attainment, smoking, group of number of remaining natural teeth and BMI group.

† Adjusted for study area, sex, age group, living arrangement, group of number of remaining natural teeth, BMI group.

## Discussion

This study explored the determinants of oral hygiene behaviour and oral health-related quality of life among community-dwelling older adults in Northern Thailand. The findings revealed that most participants demonstrated good oral hygiene behaviours, particularly in routine practices such as using fluoride toothpaste and brushing for at least 2 minutes. However, certain preventive behaviours, such as the use of dental floss, remained suboptimal. A substantial proportion of participants reported infrequent or no use of dental floss, indicating a lack of comprehensive oral hygiene practices. This finding is consistent with previous research, which has highlighted that while tooth brushing is commonly practiced, interdental cleaning methods like flossing are often neglected among older adults.^25^ Public health initiatives should focus on increasing awareness about the importance of flossing and providing resources to facilitate its practice, thereby contributing to improved oral health in the aging population.

The study revealed that older adults experienced a notable impact on various dimensions of OHRQoL, with the highest burden reported in physical pain particularly difficulty eating and oral discomfort. Functional limitations such as taste disturbances and speech difficulties were also prevalent, while psychological and social concerns, though less pronounced, reflected issues of self-image and diminished life satisfaction. These findings are aligned with global research underscoring the multidimensional burden of poor oral health among aging and underserved populations. For instance, Zaheer et al^26^ highlighted the psychosocial consequences of unmet dental needs among refugee elders in Greece, emphasising that oral health extends beyond clinical indicators to affect dignity and social functioning. Similarly, Broomhead et al^27^ demonstrated how standardised OHRQoL indicators can reveal disparities across countries, aiding public health responses.

Regarding OHL, only 24.58% of older adult participants demonstrated adequate OHL, highlighting a significant challenge in their ability to access, understand and effectively use oral health information. This deficiency in OHL was notably associated with poorer oral health behaviours, potentially due to difficulties in reading health information, accurately assessing symptoms, or making informed decisions about dental care.^7^ These findings aligned with previous research indicating that limited OHL was linked to suboptimal oral hygiene practices and might adverse oral health outcomes.^7^^,^^13^ Addressing OHL gaps through targeted educational programs is crucial to empowering older adult individuals to adopt better oral hygiene practices and consequently improve their oral health outcomes

Self-care ability emerged as a critical determinant of oral hygiene behaviours among older adults. Participants requiring assistance in daily living exhibited significantly lower oral hygiene behaviour scores, highlighting how physical or functional limitations can directly hinder routine oral hygiene practices such as brushing, flossing and regular dental visits,^28^^,^^29^ thereby necessitating external support to maintain adequate oral hygiene practices. Caregiver involvement becomes essential in this context, as caregivers can assist with daily oral hygiene tasks and ensure regular dental check-ups.^30^ Recent evidence indicates that structured caregiver training programs significantly improve the oral hygiene of dependent older adults, highlighting the value of educational and practical support interventions targeting both older adult individuals and their caregivers.^31^, ^32^, ^33^ Thus, strategies aimed at enhancing self-care abilities through comprehensive caregiver education, adaptive oral hygiene tools and tailored support systems are crucial for maintaining and improving oral health among older adult populations with diminished self-care capacity.

The use of dentures was positively associated with both oral hygiene behaviour and oral health-related quality of life among the older adult participants. Denture wearers were more likely to engage in positive oral hygiene behaviours and reported significantly lower OHIP scores, indicating fewer negative impacts on quality of life. This might reflect greater oral health awareness among denture users or improved functionality, such as enhanced chewing and speaking abilities, provided by dentures. These findings align with existing literature suggesting that well-fitting dentures can improve dietary intake, self-esteem and social participation.^34^ Previous research indicated that denture use is associated with improved oral function, potentially leading to better nutritional status and overall quality of life.^11^ These positive outcomes suggested the importance of promoting denture use among edentulous older adults to enhance their oral hygiene behaviours and overall quality of life.

There were several limitations that should be acknowledged. First, the cross-sectional design limits the ability to infer causality between identified factors and oral hygiene behaviours or OHRQoL. Longitudinal studies are needed to establish temporal relationships. Second, the use of self-reported questionnaires may introduce recall and social desirability bias, particularly in assessing oral health behaviours and OHIP. Third, although the sample was selected from both urban and rural districts, it was restricted to two subdistricts in Lampang Province, which may limit generalisability to older adult’s other regions of Thailand. Fourth, the assessment of clinical oral factors, such as the number of natural teeth and denture use, relied in part on self-examination, which may be less accurate than professional dental assessments. Lastly, unmeasured confounding factors, such as dietary habits, psychological distress and access to regular dental care, may also influence oral health outcomes but were not included in this analysis.

## Conclusion

This study indicated that oral hygiene behaviour and OHRQoL among community-dwelling older adults in Northern Thailand was significantly influenced by OHL, self-care ability and denture use. Given the low levels of adequate OHL observed, targeted educational interventions are essential to empower older adults to effectively understand and manage their oral health. Additionally, supportive measures for those with reduced self-care abilities, including caregiver education programs and adaptive hygiene tools, were critical to enhancing daily oral hygiene practices. Promoting denture use among older adults with tooth loss can further improve their overall oral functionality, dietary intake and social interactions. Future policies and interventions should integrate these elements to comprehensively address oral health disparities among older adult populations.

## Author contributions

Conceptualisation: Pechrada W, Penpatsson P, Pornnapa, Sayambhu S. Data curation: Supang W. Formal analysis: Kasama P, Sayambhu S. Investigation: Pechrada W, Sayambhu S. Methodology: Surangrat P, Sayambhu S. Supervision: Sayambhu S, Kasama P. Validation: Supa V, Patcharin K, Surangrat P. Writing – original draft: Pechrada W, Penpatsson P, Pornnapa S, Supang W, Supa V. Writing – review and editing: Patcharin K, Surangrat P, Kasama P, Sayambhu S. All authors have read and approved the final manuscript.

## Declaration of generative AI and AI-assisted technologies in the writing process

During the preparation of this manuscript, the authors used GPT-4o (OpenAI) to assist with grammar correction. All content was subsequently reviewed, edited and approved by the authors, who take full responsibility for the integrity and accuracy of the final manuscript.

## Conflict of interest

None disclosed.

## Appendix Supplementary materials

Image, application 1
