# Supplementary material for: Determinants of Oral Hygiene Behaviours and Oral Health–Related Quality of Life Among Older Adults in Northern Thailand
Source: Int Dent J. 2025 Dec 10;76(1):109314. doi: 10.1016/j.identj.2025.109314 (PMC12753216; doi:10.1016/j.identj.2025.109314)
Supplement: Supplementary file 1 [file mmc1.docx]

**Oral hygiene behavior questionnaire**

| No. | Questions | Frequency | | |
| --- | --- | --- | --- | --- |
|  |  | Never | Sometimes | Always |
| 1 | You brush your teeth for at least 2 minutes each time. |  |  |  |
| 2 | You share your toothbrush with family members or others. * |  |  |  |
| 3 | You change your toothbrush when the bristles are frayed or splayed. |  |  |  |
| 4 | You clean your teeth using dental floss. |  |  |  |
| 5 | You brush your teeth forcefully back and forth. * |  |  |  |
| 6 | You brush your teeth using toothpaste containing fluoride. |  |  |  |
| 7 | You use your teeth to open bottles, tear packaging, or cut plastic. * |  |  |  |
| 8 | When you have a toothache, you wait for the pain to subside on its own. * |  |  |  |
| 9 | When you notice cavities, decay, or tooth erosion, you do delay treatment until symptoms worsen. * |  |  |  |
| 10 | When you notice abnormalities in your mouth, you visit a dental professional. |  |  |  |
| 11 | You eat sweet, sticky snacks between meals. * |  |  |  |
| 12 | You currently smoke * |  |  |  |
| 13 | You rinse your mouth with water after consuming sweets or sugary drinks. |  |  |  |
| 14 | You drink water instead of soda, sugary drinks, tea, or coffee. |  |  |  |
| 15 | You check your gums and teeth for cleanliness and abnormalities. |  |  |  |

***** is a negative question.
